# Time window for cognitive activity involved in emotional processing

**DOI:** 10.1186/1880-6805-33-21

**Published:** 2014-07-23

**Authors:** Midori Motoi, Yuka Egashira, Takayuki Nishimura, Damee Choi, Riko Matsumoto, Shigeki Watanuki

**Affiliations:** 1 Graduate School of Integrated Frontier Sciences, Kyushu University, JSPS Research Fellow DC, 4-9-1 Shiobaru, Minami-ku, Fukuoka 815-8540, Japan; 2 Graduate School of Integrated Frontier Sciences, Kyushu University, 4-9-1, Shiobaru, Minami-ku, Fukuoka 815-8540, Japan; 3 Nagasaki University Graduate School of Biomedical Sciences, 1-12-4 Sakamoto, Nagasaki 852-8523, Japan; 4 Faculty of Design, Kyushu University, 4-9-1 Shiobaru, Minami-ku, Fukuoka 815-8540, Japan

**Keywords:** Event-related potentials, N100, Late positive potentials, BIS/BAS, Pleasant, Unpleasant

## Abstract

**Background:**

From previous studies it is becoming evident that the processing of unpleasant stimuli occurs early (0 to 300 ms); however, it is not clear how cognitive processing related to pleasant/unpleasant emotions occurs at later time windows (≥300 ms). On the other hand, as evident from the previous reports, BIS and BAS personality traits are strongly associated with unpleasant and pleasant responses, respectively. Therefore, in the present study, we aim to identify the time window involved in human pleasant/unpleasant emotional processing by investigating ERP components correlated with BIS/BAS personality traits.

**Methods:**

Twenty-nine men took part in the study and recording ERP during presented sounds. BIS/BAS score was calculated using the Japanese edition of the BIS/BAS questionnaire.

**Results:**

Significant correlation was not observed between BIS and BAS scores. A significant and positive correlation was observed between N100 amplitude and BIS score. A positive correlation was found between BAS fun seeking subscale score and LPP amplitude. Our findings did not contradict previous study results.

**Conclusions:**

Our results suggest that the processing of unpleasant emotions takes place early on, since N100 response was larger in high BIS subjects who are known to be sensitive to unpleasant emotions. LPP was larger in high BAS subjects who are known to be sensitive to pleasant emotions. The LPP was considered to be augmented because the ACC activity level during pleasant emotions reflected on LPP.

## Findings

### Background and objectives

Judgments in response to emotional stimuli have evolved in profound relation to organism survival. Emotions are generated a few milliseconds (ms) after the onset of stimuli and continue for a few seconds [1]. This process has been gradually elucidated by studies using event-related potentials (ERP) that are superior in time-resolution.

According to Olofsson, the early component of ERP (<300 ms) that is induced from unpleasant stimuli selected from the International Affective Picture System (IAPS) is larger than that from neutral or pleasant stimuli [1]. In particular, it has been reported that an early negative component in the frontal region becomes larger when a negative picture is presented [2]. In contrast, details regarding pleasant emotions are not fully understood. Mid to late ERP components (≥300 ms) are highly influenced by inherent arousing stimuli, where P300 and late positive potential (LPP) respond to both pleasant and unpleasant stimuli, thus indicating that consistent findings on the matter have not been obtained [1]. For example, although it has been reported that P300 in the frontal region increases depending on emotional valence and arousal [3], it has also been reported that arousal has a greater impact [4]. Therefore, it has been suggested that hedonic valence and arousal affect one another with complexity more than previously assumed through prior ERP studies [5].

From the above findings, it is becoming evident that the processing of unpleasant stimuli occurs early (0 to 300 ms); however, it is not clear how cognitive processing related to pleasant/unpleasant emotions occurs at later time windows (≥300 ms). Thus, by using the differences in ERP response due to personality trait, cognitive processing of emotions that are not affected by arousal, this can be clarified.

There is individual variation in stimuli response, and the differences in the individual's manner of evaluative and emotional experience is thought to contribute to the variation. Sensitivity and strength of the approach, and withdrawal systems toward stimuli cause these differences.

For many years, these two systems have been considered to control the activity of not only humans but also other animals. A connection to neural circuitry was discovered by Gray (1981) as the Behavioral Inhibition System (BIS)/Behavioral Activation System (BAS) theories [6]. Gray used this dualism in order to understand the behavior of organisms; however, humans act and behave by activating and suppressing each system with further complexity. These form different personalities, and are thought to generate the differences in evaluative and emotional experiences toward the same stimulus [7]. A questionnaire that evaluates the intensity of this system's function as a personality trait [8] was designed. BIS/BAS questionnaire has in fact been suggested for its association with various emotional responses. For example, it has been reported that subjects with high BIS scores subjectively evaluate unpleasant stimulus more unpleasantly, and subjects with high BAS scores evaluate pleasant stimulus more pleasantly [9]. In an ERP study, it has also been reported that high BIS subjects have greater N100 amplitude in response to sound stimuli during negative picture presentation, and high BAS subjects have increased P300 amplitude in response to sound stimuli during positive picture presentation [10].

As evident from the above reports, BIS and BAS are strongly associated with unpleasant and pleasant responses, respectively. Consequently, it is expected that ERP components correlated with BIS score are associated with unpleasant emotional responses, and that ERP components correlated with BAS score are associated with pleasant emotional responses. Therefore, it is postulated that the time window in which hedonic cognitive process is reflected can be clarified by identifying these ERP components.

In addition, most of the prior similar studies used visual stimuli and there are very few studies on pleasant/unpleasant responses to auditory stimuli. Thus by using short impact sounds, which have not been investigated thoroughly from a physiological perspective, as stimuli, we will elucidate an element of the response process to everyday sound. In particular, we focused on the sound of a car door closing. It is believed that the sound of a closing car door can contribute to the overall impression (pleasant-unpleasant) of the car and many publications have dealt with door closing sounds [11]. Therefore, it is thought that this sound can evoke enough emotion for it to be used as a stimulus. Additionally, a previous study using semantic differential methods shows that there does not seem to be any cultural influence on the evaluation of these sounds [12]. It is appropriate to consider the relationship between personality and emotions. We decided to use stimulus for these two points.

In summary, although the time window in which pleasant/unpleasant emotions are processed in response to everyday sound stimuli has not been elucidated, by investigating the ERP components correlated with BIS/BAS personality traits that are profoundly associated with emotional responses, it is thought that the time window that reflects the hedonic cognitive process can be clarified. Therefore, in the present study, we aim to identify the time window involved in human pleasant/unpleasant emotional processing by investigating ERP components correlated with BIS/BAS personality traits.

### Methods

#### Subjects

Twenty-nine men, aged 21 to 24 years old, without hearing impairment took part in the study. Written informed consent was obtained from the patient for the publication of this report and any accompanying images. All study protocols were approved by the ethics committee in the Department of Design at Kyushu University, Japan.

#### Presented stimulus

The impact sound that is generated when a car door is closed was used as the stimulus. Three types of car door sounds (maximum sound pressure 65 dB (A)) that were recorded outside of the car in a hemi-anechoic room were used in this study.

The temperature and humidity in the laboratory room were 26°C and 50%, respectively.

The recorded sound stimuli were digitized from the sound card installed in the computer. The output signal subsequently passed through the following equipment in order: an audio processor (SE-U55SX2, ONKYO Corporation, Osaka, Japan), a low-pass filter (NF DV8FL, cutoff frequency 20,000 Hz), a digital equalizer (dbx iEQ-31, Harman, Stanford, CT, USA), and a headphone amplifier (AT-HA20, Audio-Technica Corporation, Tokyo, Japan). The sounds were then presented to both ears of the study participants via headphones (T40RPmkIIn, FOSTEX Company, Tokyo, Japan).

#### Experimental Procedure

In each session, three types of car door closing sounds were randomly presented 60 times each at 1.5 to 2 second intervals. Three sessions were conducted with five-minute breaks in between.

#### EEG (electroencephalography) and analysis

**Figure 1:**
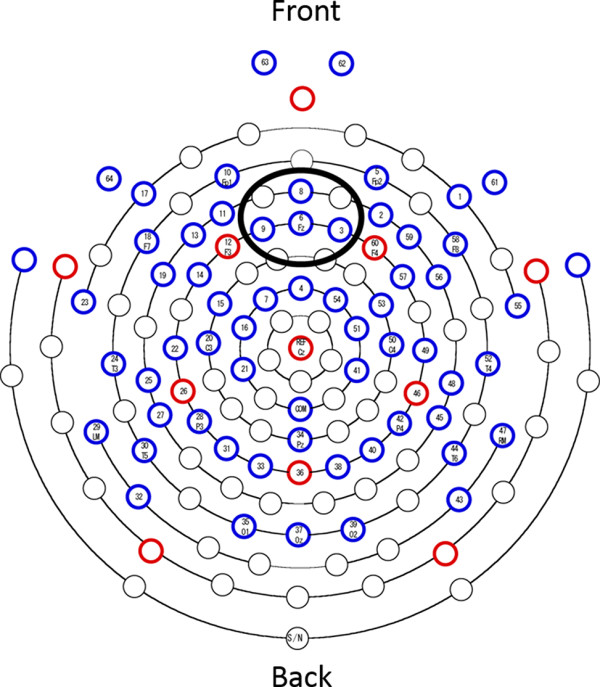
**Sensor layout and region of interesting.** The EGI 64 electrode HydroCel Geodesic Sensor Net is displayed above. Solid-line circle indicates middle frontal recording sites averaged for event-related potential (ERP) components. Electrode map is used with permission from Electrical Geodesics, Inc., Eugene, OR, USA.

Brainwaves were collected with a 64-channel net (HydroCel Geodesic Sensor Net, Electrical Geodesics, Inc., Eugene, OR, USA), amplified (Net Amps 200 64-channel EEG Amplifier, Electrical Geodesics, Inc., Eugene, OR, USA), and subsequently measured with an EEG (Net Station version 4.1.2, Electrical Geodesics, Inc., Eugene, OR, USA). Electrode resistance was maintained at ≤ 100 kΩ during the experiment, and data were continuously recorded at a sampling frequency of 500 Hz. EMSE-data editor version 5.5 was used for analysis. At that time, a bandpass filter (0.1 to 60 Hz) was used and the mean of all electrodes, excluding 61 to 64 channels, was used as the reference. Individual electrode data that were not normally recorded due to effects of noise or other reasons were excluded from the analysis. Artifact rejections were conducted manually on the basis of deflections above 60 μV from the baseline. For the remainder of the trials, stimulus presentation was set as 0 ms, and an arithmetic mean of the −100 to 800 ms range was taken to obtain the ERP waveform. Baseline correction of ERP was carried out by subtracting the mean value up to 0 ms from the overall waveform. The number of additions was set as ≥ 100 (mean: 125 times, minimum 100 to maximum 162 times, standard deviation 17.59). The mean potentials of the electrodes that were in close proximity of each other were taken by locating mid-frontal electrode sites as the region of interest (3, 6 (Fz), 8, 9, see Figure 1). With 80 to 160 ms as N1 and 450 to 800 ms as LPP, the mean value of these intervals was set as the amplitude for each ERP component. Two participants’ LPP data were removed in this analysis due to artifact on ERP waveform from 600 ms after the stimulus onset.

#### BIS/BAS score

BIS/BAS score was calculated using the Japanese edition of the BIS/BAS questionnaire [7].

#### Statistical analysis

Using R (version 3.0.3), Pearson's regression analysis was conducted with a significance level of 0.05.

### Results

**Figure 2:**
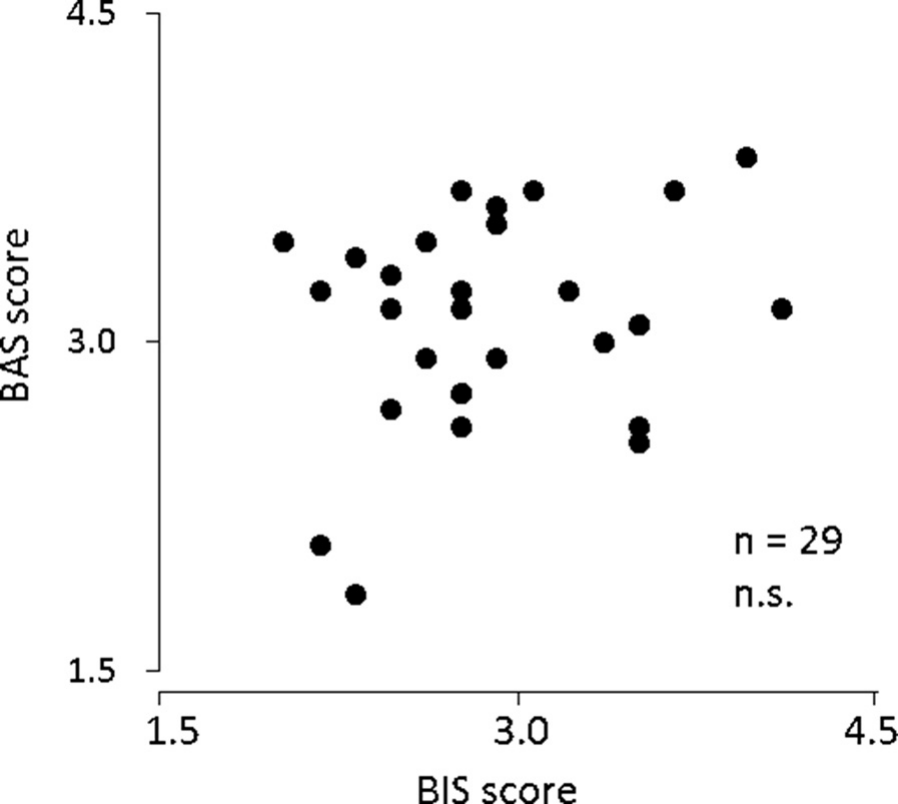
Correlation between Behavioral Inhibition System (BIS) and Behavioral Activation System (BAS).

**Figure 3:**
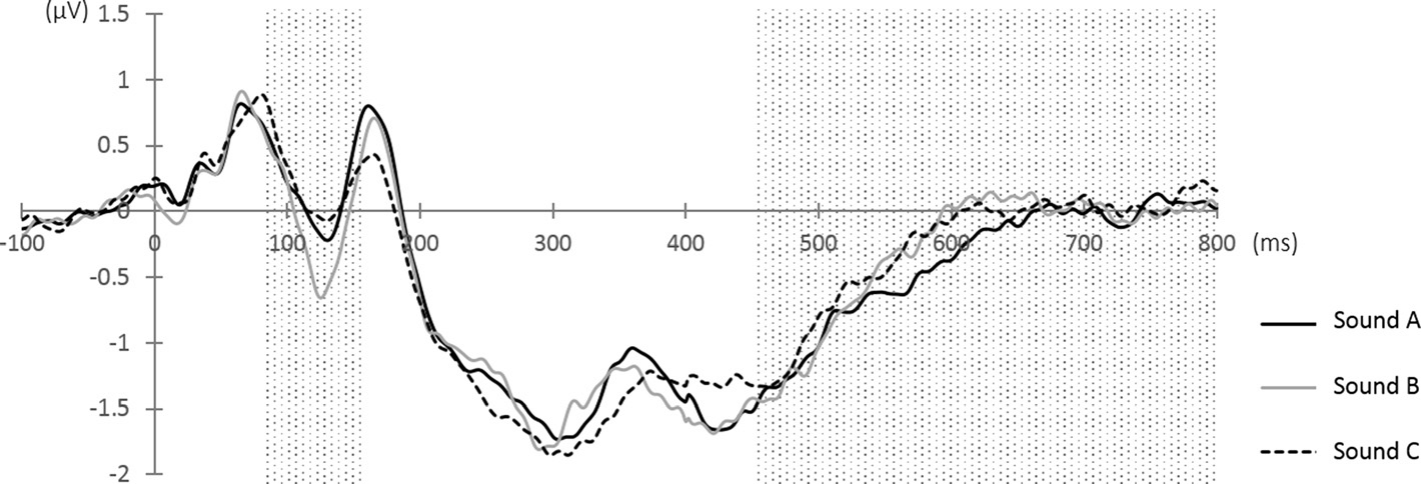
Event-related potential (ERP) wave form (middle frontal region).

The subjects' mean BIS score was 2.85 ± 0.51 and the mean BAS score was 3.10 ± 0.46. Significant correlation was not observed between BIS and BAS scores (r = 0.23, ns) (Figure 2).The ERP waveform shown in Figure 3 was obtained.

Significant and negative correlation was shown between N1 component and BIS. This means that there was a positive correlation between N1 amplitude and BIS, and thus N1 was larger in subjects with larger BIS. LPP component showed significant and positive correlations with BAS fun seeking subscale. LPP was larger in subjects with larger BAS fun seeking subscale (Table 1).

**Table 1:** **Correlations between event-related potential (ERP) and Behavioral Inhibition System**/**Behavioral Activation System (BIS/BAS)**

	**80 to 160 ms(n = 29)**	**450 to 800 ms(n = 27)**
BIS	−0.33*	−0.19
BAS	0.04	0.18
BAS-drive	0.17	0.14
BAS-Reward	−0.19	0.03
BAS-Fun seeking	0.19	0.40*

*Significant at *P* < 0.05.

### Discussion

#### BIS/BAS

Significant correlation was not observed between BIS and BAS scores (Figure 2). This showed that BIS and BAS are indices that indicate different personalities. Previous studies have also shown that BIS and BAS scores are not correlated with each other and are known to be independent factors. Thus, it can be deduced the BIS/BAS questionnaire was appropriately answered.

#### Negative component at 80 to 160 ms

A significant and positive correlation was observed between N100 amplitude and BIS score (Table 1). It was postulated that the higher BIS score, the more responsive the subjects were to unpleasant emotions evoked by sound. It has been reported that N100 becomes greater during unpleasant emotions caused by visual stimuli [10] and that BIS and N100 amplitude show a positive correlation [9]. Our results were consistent with these previous findings, and therefore suggest that the processing of unpleasant emotions takes place early at around 100 ms.

#### Late positive component at 450 to 800 ms

A positive correlation was found between BAS fun seeking subscale score and LPP amplitude (Table 1). It has been reported that the positive component P3b (439 to 630 ms), a similar time window as the LPP in the present study, becomes larger at the frontal region in response to pleasant stimuli [13]. Nonetheless, since arousal affects ERP, consistent results have not been obtained with regards to late component hedonic response. It is, therefore, difficult to simply regard that high BAS subjects had larger LPP response because they felt stronger pleasant emotions.

Nonetheless, in the present study, only the BAS fun seeking subscale showed correlation with LPP. In a previous study that used ERP, only the BAS fun seeking subscale especially showed correlation with Pe (positive potential that appears at 164 to 360 ms), suggesting the association with the activity level in the anterior cingulate cortex (ACC) [14]. The ACC is a part of the brain that is involved in proactive motivation, and is also considered to be associated with pleasant emotions [15]. As above, our findings suggest that processing of unpleasant emotions takes place early at around 100 ms, and that processing of pleasant emotions occurs after 450 ms for human emotional response. Processing related to early automatic attention is functionally active for stimuli that could be a negative threat, and the responses to positive stimuli follow. Such responses are considered to arise from the biological significance of the stimuli [1,16].

#### Limitations of the present study

We did not use the sound stimuli set of International Affective Digitized Sounds that were often used to evoke emotions as in previous studies [17]. Since early components are particularly known to be strongly influenced by the intensity of the stimulus, we were considerate and mindful of stimuli intensities such as volume adjustments.

### Conclusion

Our results suggest that the processing of unpleasant emotions takes place early on, since N100 response was larger in high BIS subjects who are known to be sensitive to unpleasant emotions. Our findings did not contradict previous study results.

LPP was larger in high BAS subjects who are known to be sensitive to pleasant emotions. The LPP was considered augmented due to the activity level in the ACC. The association of pleasant emotions was suggested as a late component response, a time window that was not previously viewed with consistency.

## Abbreviations

ACC: anterior cingulate cortex; BAS: Behavioral Activation System; BIS: Behavioral Inhibition System; ERP: event-related potential; LPP: late positive potential.

## Competing interests

The authors declare that they have no competing interests.

## Authors’ contributions

MM and SW contributed to the design of the experiment. MM performed the experiments, analyzed the data and wrote the manuscript with advice of SW. TN, DC, and YE participated in the discussion and preparation of the manuscript. RM helped coordinate research activities. All authors read and approved the final manuscript.
